# Urinary albumin-to-creatinine ratio as an independent predictor of long-term mortality in atherosclerotic cardiovascular disease patients: A propensity score-matched study: UACR and Long-term Mortality in ASCVD

**DOI:** 10.1016/j.ajpc.2024.100920

**Published:** 2024-12-18

**Authors:** Houyong Zhu, Chao Yang, Xiao Liu, Xiaoqun Xu, Qilan Chen, Xiaojiang Fang, Jinyu Huang, Tielong Chen

**Affiliations:** a Department of Cardiology, Hangzhou TCM Hospital Affiliated to Zhejiang Chinese Medical University, Hangzhou, Zhejiang, PR China; b The Fourth School of Clinical Medicine, Zhejiang Chinese Medical University, Hangzhou, Zhejiang, PR China; c Hangzhou Red Cross Hospital, Hangzhou, Zhejiang, PR China; d Department of Cardiology, Hangzhou First People's Hospital, Hangzhou, Zhejiang, PR China

**Keywords:** Atherosclerotic cardiovascular disease, Urinary albumin‒creatinine ratio, All-cause mortality, Propensity score matching

## Abstract

**Background and Aims:**

Atherosclerotic cardiovascular disease (ASCVD) is a leading cause of mortality, and while the association between the urinary albumin-to-creatinine ratio (UACR) and cardiovascular risk is recognized, the specific impact of UACR on the long-term survival of ASCVD patients remains not fully understood. The aim of this study is to investigate the influence of UACR on the long-term risk of all-cause mortality in patients with ASCVD.

**Methods:**

This study included ASCVD patients from the National Health and Nutrition Examination Survey (NHANES) from 1999 to 2018. Mortality outcomes were ascertained by linkage to the National Death Index as of December 31, 2019. UACR risk was stratified into three levels: Group 0 (UACR < 30 mg/g), Group 1 (30–300 mg/g), and Group 2 (>300 mg/g). The primary outcome was all-cause mortality, with cardiovascular mortality as a secondary outcome. Cox proportional hazards, adjusted for demographic factors, traditional cardiovascular risk factors, and secondary prevention medications for ASCVD, were used to analyze the cumulative risk of outcomes. Propensity score matching was employed for risk adjustment, and sensitivity analyses were conducted based on cohorts with chronic coronary syndrome (CCS), stroke, heart failure, and non-heart failure.

**Results:**

Among the 1,737 patients with a median follow-up of 10 years, 1,026 all-cause deaths and 351 cardiovascular deaths were recorded. After full model adjustment, higher UACR levels were associated with increased risks of all-cause mortality (Group 1: hazard ratio (HR), 1.601; 95 % confidence interval (CI), 1.382–1.855; Group 2: HR, 2.378; 95 % CI, 1.884–3.001; both *P* < 0.001 for trend) and cardiovascular mortality (Group 1: HR, 2.080; 95 % CI, 1.631–2.652; Group 2: HR, 2.883; 95 % CI, 1.951–4.260; both *P* < 0.001 for trend). Propensity score matching confirmed these findings, showing significantly elevated all-cause mortality risks in high-risk UACR groups (with a cutoff of 30 mg/g: HR, 1.468 (95 %CI, 1.254–1.719), *P* < 0.001; with a cutoff of 300 mg/g: HR, 1.935 (95 %CI, 1.399–2.675), *P* < 0.001). All sensitivity analyses were consistent with the results of the overall cohort.

**Conclusion:**

UACR is an important prognostic indicator for predicting the long-term outcomes of ASCVD patients, with its impact being independent of eGFR.

## Graphical abstract

**Figure:**
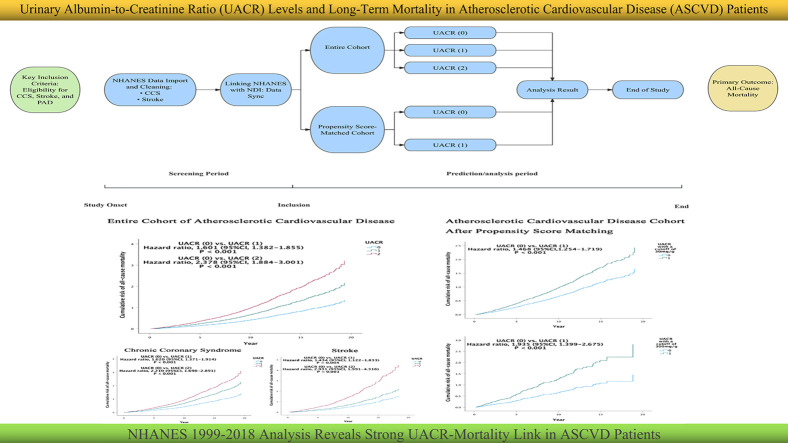

## 1 Introduction

Atherosclerotic cardiovascular disease (ASCVD) is a chronic condition caused by the accumulation of plaques composed of fat, cholesterol, and other substances within the arterial walls. ASCVD mainly includes coronary artery disease, stroke, and peripheral arterial disease, among others. Although the prevalence of secondary prevention strategies for ASCVD has led to a significant number of patients receiving treatments that have been proven effective, the incidence of ASCVD continues to rise globally due to the aging of the world's population and changes in lifestyle. It remains one of the leading causes of death worldwide. According to the World Health Organization (WHO), cardiovascular disease accounts for 33 % of all deaths globally, with ischemic heart disease (9.1 million deaths) and stroke (6.6 million deaths) making up 85 % of all cardiovascular disease deaths [1]. This indicates that the mortality rate of ASCVD continues to be a significant public health issue. The optimization of secondary prevention strategies is an urgent issue to address. In line with the widely accepted principles for developing secondary prevention strategies for ASCVD, the key lies in effectively assessing and managing the risks in patients who have already been diagnosed with ASCVD, particularly in identifying those at high-risk status. While current prevention principles are primarily based on risk factors such as blood lipids, thrombotic risk, blood pressure, and diabetes [2], the control and monitoring of other factors that may indicate disease progression, such as proteinuria, are often overlooked.

Despite previous studies confirming the association between microalbuminuria and the occurrence of atherosclerosis [3], as well as its link to an increased risk of adverse cardiovascular events, particularly among patients with poor cardiovascular health [[4], [5]], there is still a scarcity of research on the impact of microalbuminuria on the long-term prognosis of ASCVD patients. Therefore, in this study, we analyzed data from National Health and Nutrition Examination Survey (NHANES) from 1999 to 2018 and linked it with the National Death Index as of December 31, 2019, aiming to assess the impact of creatinine-adjusted urinary albumin, the urinary albumin-to-creatinine ratio (UACR), on the long-term all-cause mortality risk in ASCVD patients. We hypothesize that UACR is an independent prognostic predictor for ASCVD patients, and that this effect is independent of traditional cardiovascular risk factors.

## 2 Materials and methods

### 2.1 National health and nutrition examination survey

The NHANES is a large-scale, multi-stage, nationally representative survey conducted by the National Center for Health Statistics (NCHS) on the non-institutionalized civilian population in the United States. Since 1999, it has become a continuous program with each cycle representing a 2-year period. Each survey participant completes a home interview and undergoes a physical examination at a mobile examination center. A detailed description of the NHANES methodology is published elsewhere [[6], [7]]. NHANES has been approved by the Institutional Review Board and includes written informed consent. More detailed information could be found at www.cdc.gov/nchs/nhanes/irba98.htm.

This study collected data from ten cycles of NHANES (1999–2018). The ASCVD patients included in this study comprised individuals with chronic coronary syndrome (CCS) and stroke. The diagnosis of CCS referred to the 2019 ESC Guidelines for Chronic Coronary Syndromes [8], and the history of stroke was identified through a combination of self-reported physician diagnoses and standardized medical condition questionnaires administered during personal interviews, with specific diagnostic criteria detailed in Appendix 1. After excluding participants who did not meet the criteria, 2201 participants with ASCVD were included as subjects of the study. After excluding 462 participants with missing creatinine and urinary albumin, 1739 participants remained. By linking with the National Death Index as of December 31, 2019, the death status of these participants was determined, including cardiovascular mortality and all-cause mortality [9]. Among the 1739 participants, the death status of 2 individuals was disqualified, leaving a final analysis of 1737 patients (Supplementary Figure 1).

### 2.2 Baseline data

Information on age, gender, race, education level, smoking status, drinking status, and the poverty income ratio (PIR) was collected using standardized questionnaires from home interviews. PIR was calculated by dividing the family income by the poverty threshold specific to the family size, as well as the appropriate year and state. Body weight and height were obtained from physical examinations, and the body mass index (BMI) was calculated as weight divided by the square of height. Race was categorized into non-Hispanic white, non-Hispanic black, Hispanic-Mexican American, and other. The “other” category includes individuals who identify as Hispanic but not Mexican American, as well as those who identify as a race other than white or black, or those who select multiple races. Education level was divided into less than high school and high school and above. Biochemical indicators were obtained from the biochemical profiles of NHANES laboratory examinations. The estimated glomerular filtration rate (eGFR) was calculated using the Chronic Kidney Disease Epidemiology Collaboration equation recommended by Inker et al., based on creatinine [10]. Through questionnaires, we also obtained medical histories such as diabetes, hypertension, cancer, abnormal liver function, and family history of cardiovascular diseases (CVD), with specific diagnostic criteria detailed in Appendix 1. The measurement of UACR-related indicators included urine samples; briefly, urinary albumin was measured by solid-phase fluorescence immunoassay, and urinary creatinine was measured by enzymatic methods [11].

### 2.3 Risk stratification of UACR

According to the Kidney Disease: Improving Global Outcomes (KDIGO) guidelines [12], the risk stratification of UACR is divided into three levels from low to high: Group 0 with UACR <30 mg/g, Group 1 with UACR between 30 and 300 mg/g, and Group 2 with UACR greater than 300 mg/g.

### 2.4 Outcomes

We utilized death certificate information provided by the National Death Index (NDI) up to December 31, 2019. Matching with NHANES and NDI was determined by identifying a unique individual sequence number (SEQN). The primary outcome was all-cause mortality, defined as death from any cause. The secondary outcome was cardiovascular mortality. The main cause of death was classified according to the International Statistical Classification of Diseases and Related Health Problems, 10th Revision (ICD-10), and the standardized code list (UCOD_LEADING) created by the National Center for Health Statistics (NCHS). The cardiovascular mortality code is 001, which includes 15 causes of cardiovascular death, such as ischemic heart disease, hypertensive heart disease, and myocarditis. For more information, please visit https://www.cdc.gov/nchs/data/datalinkage/public-use-linked-mortality-files-data-dictionary.pdf.

### 2.5 Statistical analysis

We investigated the baseline characteristics across different UACR risk levels using the following methods: continuous variables were summarized with medians and interquartile ranges and tested using the Kruskal–Wallis test, while categorical variables were assessed using the Pearson chi-square test. Missing values for continuous variables were imputed using the Expectation-Maximization (EM) method, and missing categorical variables were filled by adding a missing value category. Based on our assessment of the potential for confounding variables in the relationship between UACR and outcome events, survival analysis was estimated using the Cox proportional hazards model with an inclusive model. The relevant confounding variables adjusted for included demographic factors (age, gender, race, education level, marital status, and PIR), traditional cardiovascular risk factors (smoking, alcohol use, BMI, total cholesterol, diabetes, hypertension, chronic lung disease, abnormal liver function, cancer, family history of cardiovascular disease, estimated glomerular filtration rate (eGFR)), and secondary prevention medications for ASCVD (angiotensin-converting enzyme inhibitor (ACEI)/ angiotensin receptor blocker (ARB), beta-blocker, lipid-lowering therapy, antiplatelet aggregation therapy, and calcium-channel blocker). Model 1 was adjusted for basic demographic factors; Model 2 was adjusted for the variables of Model 1 plus traditional cardiovascular risk factors; Model 3 was adjusted for the variables of Model 2 plus the aforementioned secondary prevention medications for ASCVD, serving as our fully adjusted model. Hazard ratios (HR) and their corresponding 95 % confidence intervals (CI) were obtained from the Cox proportional hazards Model 3, and a standard graph of cumulative risks for outcomes was established based on this model. The absence of time-varying effects for UACR was confirmed by visually assessing the standard graph of the negative logarithm of the negative logarithm of the Cox survival function against cumulative risk, thereby validating the assumptions of the Cox model. Propensity score matching was performed to adjust for baseline data imbalances between UACR groups.

Propensity scores were generated for each patient using UACR as the dependent variable and all variables from the baseline data as independent variables, and the C-statistic was calculated to assess the robustness of the propensity score matching. The “nearest neighbor” matching method was used (with a fixed caliper width of 0.2), to match the patients' propensity scores on a 1:1 basis without replacement. After matching, standardized differences were used, with general balance reflected by standardized differences <25 %, and high balance reflected by standardized differences <10 % [13]. Following the matching, a univariate Cox proportional hazards model was employed to obtain HR and the corresponding 95 % CI.

We conducted subgroup analyses based on age, gender, race, education level, smoking, drinking, hypertension, chronic lung disease, BMI, diabetes, cancer, liver dysfunction, eGFR, family history of CVD, and follow-up time in the fully adjusted model (Model 3). Additionally, to further exclude interference with the outcomes, we conducted sensitivity analyses on cohorts with CCS, stroke, heart failure, non-heart failure, and those already undergoing secondary prevention, respectively. HR values (95 % CI), percentages, medians (interquartile ranges) were used as summary statistics in the corresponding cases. A two-sided P-value <0.05 was considered statistically significant. Data were analyzed using SPSS 27.0 (SPSS, Inc., Chicago, IL).

## 3 Results

### 3.1 Patient characteristics

A total of 462 patients missing blood or urine creatinine or urine protein, along with 2 patients with ineligible death status, were excluded. Ultimately, 1737 ASCVD subjects were included in this study, with 1026 all-cause deaths and 351 cardiovascular deaths recorded. A comparison table of baseline characteristics between excluded and included patients is presented in Supplementary Table 1. Among the included patients, Ages ranged from 20 to 85 years, with a median age of 70 years; 42.4 % were female, 62.6 % were non-Hispanic whites, and the median follow-up time was 10 years (Table 1). There were 577 (33.2 %) with diabetes, 1404 (80.8 %) with hypertension, 464 (26.7 %) with chronic lung disease, 104 (5.9 %) with abnormal liver function, 351 (20.2 %) with cancer, and 249 (14.3 %) with a family history of CVD. Additionally, 775 (44.6 %) patients regularly took ACEI/ARB, 759 (43.6 %) regularly took beta-blockers, 810 (46.6 %) regularly underwent lipid-lowering therapy, 256 (14.7 %) regularly took antiplatelet aggregation therapy, and 285 (16.9 %) regularly took calcium-channel blockers. Missing values were imputed using the EM method. Missing data were present in the following variables: PIR, BMI, education status, marriage, smoking, alcohol use, hypertension, chronic lung disease, family history of CVD, antiplatelet aggregation therapy, beta-blocker, ACEI or ARB, and lipid lowering therapy. The corresponding missing rates were 7.1 %, 4.8 %, 0.1 %, 0.8 %, 38.5 %, 18.8 %, 0.1 %, 0.1 %, 38.5 %, 8.8 %, 8.8 %, 8.5 %, and 8.4 %, respectively. For further details, refer to Supplementary Table 2.

**Table 1:** Baseline Characteristics by UACR Levels in NHANES 1999–2018.

Vairable	UACR, mg/g			
	0 (<30) (*N* = 1257)	1 (30–300) (*N* = 360)	2 (>300) (*N* = 120)	P Value
Age, median (quartile), years	68 (58–78)	74 (65–81)	68.5 (61–80)	<0.001
Gender (Female), no. (%)	548 (43.6)	147 (40.8)	43 (35.8)	0.201
Race/ethnicity, no. (%)				0.010
Hispanic-Mexican American	154 (12.3)	44 (12.2)	26 (21.7)	
Non-Hispanic White	798 (63.5)	230 (63.9)	60 (50)	
Non-Hispanic Black	215 (17.1)	67 (18.6)	29 (24.2)	
Other*	90 (7.2)	19 (5.3)	5 (4.2)	
Education status, no. (%)				<0.001
< High school	447 (35.6)	164 (45.6)	50 (41.7)	
≥ High school	809 (64.4)	196 (54.4)	69 (57.5)	
Marriage, no. (%)	725 (57.7)	176 (48.9)	59 (49.2)	0.016
Smoking, no. (%)				0.415
Never	32 (2.5)	6 (1.7)	4 (3.3)	
Current	207 (16.5)	45 (12.5)	17 (14.2)	
Former	545 (43.4)	165 (45.8)	48 (40)	
Alcohol use, no. (%)	248 (19.7)	58 (16.1)	27 (22.5)	0.352
PIR, median (quartile)	2.05 (1.17–3.46)	1.89 (1.18–2.63)	1.92 (1.10–2.68)	0.073
BMI, median (quartile), kg/m^2^	28.50 (25.52–32.53)	29.04 (25.26–32.92)	28.13 (25.32–32.25)	0.571
Tch, median (quartile), mmol/L	4.84 (4.11–5.66)	4.77 (4.01–5.61)	5.07 (4.3–6.01)	0.035
Diabetes, no. (%)	342 (27.2)	156 (43.3)	79 (65.8)	<0.001
Hypertension, no. (%)	995 (79.2)	302 (83.9)	107 (89.2)	0.007
Chronic lung disease, no. (%)	350 (27.8)	88 (24.4)	26 (21.7)	0.002
Liver dysfunction, no. (%)	70 (5.6)	28 (7.8)	6 (5)	0.266
Cancer, no. (%)	245 (19.5)	80 (22.2)	26 (21.7)	0.626
Family history of CVD, no. (%)	186 (14.8)	51 (14.2)	12 (10)	0.079
eGFR (mL/min/1.73m^2^), no. (%)				<0.001
>90	383 (30.5)	69 (19.2)	20 (16.7)	
60–90	579 (46.1)	156 (43.3)	28 (23.3)	
30–60	278 (22.1)	114 (31.7)	51 (42.5)	
15–30	15 (1.2)	18 (5.0)	17 (14.2)	
<30	2 (0.2)	3 (0.8)	4 (3.3)	
Medication, no. (%)				
ACEI or ARB	544 (43.3)	164 (45.6)	67 (55.8)	<0.001
Beta-blocker	525 (41.8)	166 (46.1)	68 (56.7)	<0.001
Lipid lowering therapy	598 (47.6)	154 (42.8)	58 (48.3)	<0.001
Antiplatelet aggregation therapy	191 (15.2)	44 (12.2)	21 (17.5)	<0.001
Calcium-channel blocker	186 (14.8)	80 (22.2)	29 (24.2)	<0.001

⁎ This means “Other” encompasses both “Other Hispanic” and “Other Non-Hispanic Races”. “Other Hispanic” refers to individuals who self-identify as Hispanic but are not of Mexican American descent. “Other Non-Hispanic Races” pertains to individuals who self-identify as a race other than white or black, or those who select multiple races. ACEI: angiotensin-converting enzyme inhibitor; ARB: angiotensin receptor blocker; BMI: body mass index; CVD: cardiovascular disease; eGFR: estimated glomerular filtration rate; PIR: poverty income ratio; Tch: total cholesterol; UACR: urinary albumin-to-creatinine ratio. Values are numbers (%) or medians (quartile).

### 3.2 Outcomes for the entire study cohort

#### 3.2.1 All-Cause mortality

**Fig. 1:**
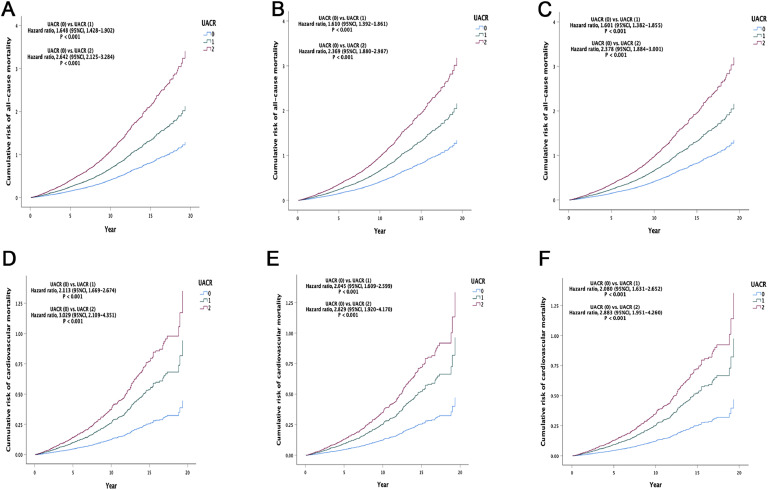
Cumulative Incidence of All-Cause and Cardiovascular Mortality Across UACR Levels in Different Models. A. Model 1: All-Cause Mortality Cumulative Incidence for UACR-Adjusted for Basic Demographics. B. Model 2: All-Cause Mortality Cumulative Incidence for UACR-Adjusted for Demographics Plus Traditional Cardiovascular Risk Factors. C. Model 3: All-Cause Mortality Cumulative Incidence for UACR-Fully Adjusted Model Including Secondary Prevention Medications for ASCVD. D. Model 1: Cardiovascular Mortality Cumulative Incidence for UACR-Adjusted for Basic Demographics. E. Model 2: Cardiovascular Mortality Cumulative Incidence for UACR-Adjusted for Demographics Plus Traditional Cardiovascular Risk Factors. F. Model 3: Cardiovascular Mortality Cumulative Incidence for UACR-Fully Adjusted Model Including Secondary Prevention Medications for ASCVD. Model 1 adjustments include basic demographic factors; Model 2 builds upon Model 1 with the addition of traditional cardiovascular risk factors; Model 3 is the fully adjusted model that incorporates variables from Model 2 as well as secondary prevention medications for ASCVD. ASCVD: atherosclerotic cardiovascular disease; UACR: urinary albumin-to-creatinine ratio.

In the fully adjusted model (Model 3), compared to the UACR (Group 0), the risk of all-cause mortality was higher for UACR (Group 1) [HR, 1.601 (95 %CI, 1.382–1.855), *P* < 0.001] and UACR (Group 2) [HR, 2.378 (95 %CI, 1.884–3.001), *P* < 0.001], and the risk of all-cause mortality increased with higher levels of UACR (*P* < 0.001 for trend). The results of Model 3 were consistent with those of the other two models (Model 1 and 2) (Table 2 and Figs. 1A-C).

**Table 2:** Cox Regression Analysis for UACR Predictions of Outcomes.

Outcomes	UACR, HR (95 % Cl), mg/g			
	0 (<30) (*N* = 1257)	1 (30–300) (*N* = 360)	2 (>300) (*N* = 120)	P for trend
All-cause mortality				
Model 1	1.000 (Reference)	1.648 (1.428–1.902)	2.642 (2.125–3.284)	<0.001
P Value		<0.001	<0.001	
Model 2	1.000 (Reference)	1.610 (1.392–1.861)	2.369 (1.880–2.987)	<0.001
P Value		<0.001	<0.001	
Model 3	1.000 (Reference)	1.601 (1.382–1.855)	2.378 (1.884–3.001)	<0.001
P Value		<0.001	<0.001	
Cardiovascular mortality				
Model 1	1.000 (Reference)	2.113 (1.669–2.674)	3.029 (2.109–4.351)	<0.001
P Value		<0.001	<0.001	
Model 2	1.000 (Reference)	2.045 (1.609–2.599)	2.829 (1.920–4.170)	<0.001
P Value		<0.001	<0.001	
Model 3	1.000 (Reference)	2.080 (1.631–2.652)	2.883 (1.951–4.260)	<0.001
P Value	0.001	<0.001	<0.001	

Model 1 is adjusted for age, gender, race, education status, marriage, and poverty-income ratio. Model 2 is adjusted for variables in Model 1 + smoking, alcohol use, body mass index, total cholesterol, diabetes, hypertension, chronic lung disease, liver dysfunction, cancer, family history of CVD, and estimated glomerular filtration rate. Model 3 is adjusted for variables in Model 2 + angiotensin-converting enzyme inhibitor/angiotensin receptor blocker, beta-blocker, lipid lowering therapy, antiplatelet aggregation therapy, and calcium-channel blocker. CI: confidence interval; UACR: urinary albumin-to-creatinine ratio, HR: hazard ratio.

#### 3.2.2 Cardiovascular mortality

In the fully adjusted model (Model 3), compared to the UACR (Group 0), the risk of cardiovascular mortality was higher for UACR (Group 1) [HR, 2.080 (95 %CI, 1.631–2.652), *P* < 0.001] and UACR (Group 2) [HR, 2.883 (95 %CI, 1.951–4.260), *P* < 0.001], and the risk of cardiovascular mortality increased with higher levels of UACR (*P* < 0.001 for trend). The results of Model 3 were consistent with those of the other two models (Model 1 and 2) (Table 2 and Figs. 1D-F).

#### 3.2.3 Propensity score-matched patient characteristics

When UACR was grouped using a cutoff of 30 mg/g, the propensity score model had good predictive ability for all-cause mortality (C-statistic of 0.813 for all-cause mortality), with a matching rate of 92.5 %. All baseline variables achieved a high level of balance after matching (Supplementary Table 3). Similarly, when a cutoff of 300 mg/g for UACR was used for grouping, the propensity score model also performed well (C-statistic of 0.848 for all-cause mortality), with matching rates of 90.8 %. Gender, education status, smoking, alcohol use, total cholesterol, and ACEI or ARB reached a general level of balance, while the rest of the baseline variables achieved a high level of balance after matching (Supplementary Table 3).

#### 3.2.4 Outcomes after propensity score matching

**Fig. 2:**
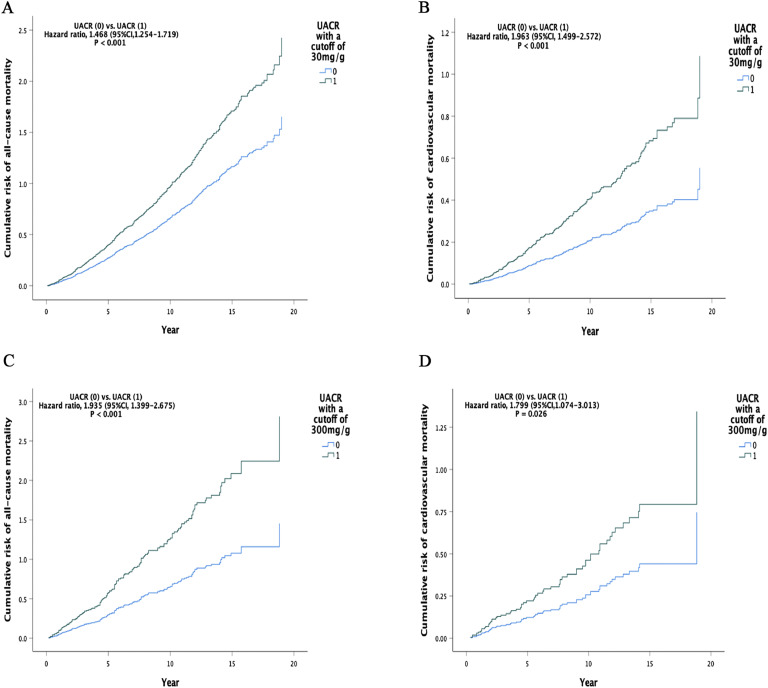
Cumulative Incidence of All-Cause and Cardiovascular Mortality by UACR Levels in Propensity Score-Matched Patient Pairs. A. Cumulative risk of all-cause mortality for UACR with a threshold of 30 mg/g. B. Cumulative risk of cardiovascular mortality for UACR with a threshold of 30 mg/g. C. Cumulative risk of all-cause mortality for UACR with a threshold of 300 mg/g. D. Cumulative risk of cardiovascular mortality for UACR with a threshold of 300 mg/g. UACR: urinary albumin-to-creatinine ratio.

After propensity score matching, in the group categorized by the UACR cutoff of 30 mg/g, compared to the low-risk (Group 0), the high-risk (Group 1) had a significantly higher risk of all-cause mortality [HR, 1.468 (95 %CI, 1.254–1.719), *P* < 0.001] and cardiovascular mortality [HR, 1.963 (95 %CI, 1.499–2.572, *P* < 0.001] (Figs. 2A and B and Supplementary Table 4). In the group categorized by the UACR cutoff of 300 mg/g, compared to the low-risk (Group 0), the high-risk (Group 1) had a significantly higher risk of all-cause mortality [HR, 1.935 (95 %CI, 1.399–2.675), *P* < 0.001] and cardiovascular mortality [HR, 1.799 (95 %CI, 1.074–3.013), *P* = 0.026] (Figs. 2C and D and Supplementary Table 4).

#### 3.2.5 Subgroup analyses for the entire cohort

**Fig. 3:**
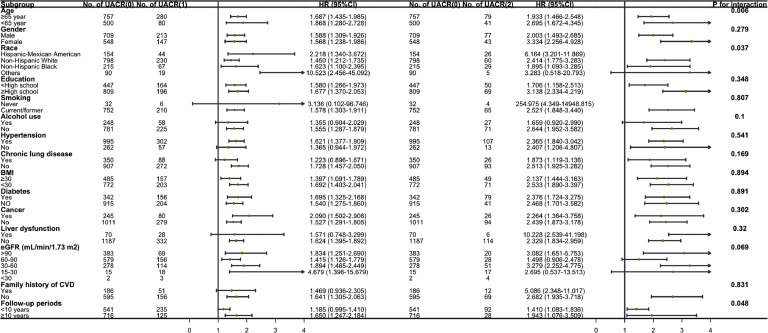
Subgroup Analyses for the All-Cause Mortality Across UACR Levels. BMI: body mass index; CVD: cardiovascular disease; eGFR: estimated glomerular filtration rate; UACR: urinary albumin-to-creatinine ratio.

The impact of UACR on all-cause mortality is consistent across subgroups defined by gender, education level, smoking, drinking, hypertension, chronic lung disease, BMI, diabetes, cancer, abnormal liver function, eGFR, and family history of CVD, with the exception of age, race, and follow-up time (P for interaction=0.006, 0.037, and 0.048, respectively) (Fig. 3). The effect of UACR on cardiovascular mortality is consistent across all subgroups (Supplementary Figure 2).

#### 3.2.6 Sensitivity analyses for the entire cohort

Regardless of whether in the CCS, stroke, heart failure, non-heart failure, or those already undergoing secondary prevention, compared to the UACR (Group 0), the risk of all-cause mortality and cardiovascular mortality increased for both UACR (Group 1) and UACR (Group 2), and the incidence of all outcome events also increased with higher levels of UACR (all *P* < 0.001 for trend) (Supplementary Table 5).

## 4 Discussion

In this nationally representative cohort study of the ASCVD population, after adequate adjustment for confounding factors, UACR levels were associated with the risk of all-cause mortality, with an increasing risk as the level of UACR stratification increased. After propensity score matching, UACR levels remained associated with the risk of all-cause mortality. The results of sensitivity analyses in the cohorts of CCS, stroke, heart failure, non-heart failure, and those already undergoing secondary prevention were consistent with the overall cohort.

UACR is an important indicator of kidney damage and, together with eGFR, forms the KDIGO risk stratification [14]. Due to the frequent occurrence of cardiorenal syndrome in clinical practice, eGFR is widely recognized as an independent risk factor for adverse cardiovascular events, but the importance of UACR is still not given enough attention. Recently, the Chronic Kidney Disease (CKD) Prognosis Consortium published a meta-analysis of individual participant data estimating the relationship between glomerular filtration rate, albuminuria, and adverse outcomes. They found that even a mild increase in UACR (30–299 mg/g) is associated with an increased risk of various adverse events, including cardiovascular adverse events, and as UACR gradually increases, its correlation with the risk of adverse events also becomes independent of eGFR [4]. Our subgroup analysis of eGFR also indicates that the impact of UACR on outcomes is not limited by the risk associated with eGFR, suggesting that it is insufficient for cardiovascular physicians to focus solely on eGFR when assessing the impact of kidney function on cardiovascular risk. In a study from the Atherosclerosis Risk in Communities (ARIC) study involving 838 ASCVD patients, it was shown that UACR greater than or equal to 30 mg/g is associated with an increased risk of major adverse cardiovascular events [15]. Additionally, two recent cohort studies, one including 2832 patients with ASCVD and diabetes, showed that patients with abnormal UACR had more than double the risk of cardiovascular mortality compared to those with normal UACR levels [16]. The other study, with 19,340 participants, also demonstrated that abnormal UACR is associated with an increased risk of cardiovascular disease, regardless of gender [17]. However, to our knowledge, there has never been a prospective study investigating the impact of UACR on the long-term mortality risk in ASCVD patients, and propensity score-matched studies, such as this one, though less powerful, provide an alternative method to determine the impact of UACR on the long-term mortality risk in ASCVD patients. Our study found that across the entire cohort, increased UACR is associated with cardiovascular death and all-cause mortality, with an increasing risk as the level of UACR stratification rises. After propensity score matching, UACR, whether mildly elevated (>30 mg/g) or severely elevated (>300 mg/g), remained consistent with the overall cohort.

The patients with ASCVD included in this study were CCS and stroke. Some studies have demonstrated a strong correlation between albuminuria and the presence or progression of CCS. Compared to individuals with normal levels of albuminuria, those with albuminuria tend to have a more severe form of CCS [18], and some research has even indicated that the risk associated with albuminuria is comparable to that of a history of myocardial infarction [19]. A meta-analysis [20] that included 38 studies with 1.7 million participants and another meta-analysis [21] that included 7 studies with 150,000 participants both showed an association between albuminuria and the incidence of stroke. Our sensitivity analysis based on cohorts with CCS and stroke yielded results consistent with the overall cohort, confirming the robustness of our findings.

Although heart failure was not the focus of this study, we acknowledge the heightened interest in UACR among this population [22] [23], especially with the emergence of sodium-dependent glucose transporters 2 (SGLT2) inhibitors in treatment regimens for heart failure and kidney disease. Evidence suggests that SGLT2 inhibitors not only lower the renal glucose threshold and improve volume status to alleviate heart failure but also reduce the risk of cardiovascular events, as confirmed in the DAPA-CKD [24], EMPA-KIDNEY [25], and CREDENCE [26] trials. Therefore, our study included sensitivity analyses with patients with and without heart failure, and the results indicated that the impact of UACR on outcomes was consistent regardless of the presence of heart failure, suggesting that the influence of UACR on ASCVD may be independent of heart failure status. According to the recently updated KDIGO guidelines [27] for the management of chronic kidney disease, SGLT2 inhibitors are strongly recommended for the treatment of CKD patients with UACR >200 mg/g. Provenzano et al. found that the combination of SGLT2 inhibitors with the mineralocorticoid receptor antagonist eplerenone can further reduce UACR in CKD patients [28]. In conjunction with the subgroup analysis of this study, which suggests that the impact of UACR on outcomes is not affected by CKD staging, future interventions could explore these therapies even in ASCVD patients but without CKD or heart failure. It is worth mentioning that the recently published EMPACT-MI trial [29] indicated that among patients after acute myocardial infarction, treatment with empagliflozin did not significantly reduce the risk of major adverse cardiovascular events compared to placebo. This may be related to some cardiac causes after acute myocardial infarction (such as stent thrombosis, recurrent myocardial infarction, mechanical complications, and scar-related ventricular arrhythmias) and non-cardiac causes within the first 30 days, which cannot be altered by SGLT2 inhibitors. Additionally, in the sensitivity analysis of the group that had already undergone lipid-lowering therapy and was on at least one other form of secondary prevention, we still found that UACR was associated with mortality, further enhancing the robustness of our study's results.

The strengths of this study are that it is the first to use propensity score matching to investigate the impact of UACR on the long-term mortality risk of ASCVD patients. The combined results from the overall cohort and the propensity score-matched cohort indicate that UACR is an important prognostic indicator for ASCVD patients, and this impact is independent of eGFR and not affected by heart failure. However, this clinical study has some limitations.The heart failure cohort was collected by NHANES staff or collaborators, and peripheral artery disease is not included in the database. Additionally, the focus of this study was to compare the impact of baseline UACR on the prognosis of ASCVD patients, without dynamically assessing changes in UACR levels. Moreover, the imbalance in baseline data between excluded and included patients, coupled with the high rate of missing data on smoking and family history of cardiovascular disease among included patients, reflects inherent limitations of retrospective studies. To address these specific challenges, we implemented measures such as missing value imputation, subgroup analysis, and propensity score matching.

## 5 Conclusions

UACR stands out as a critical prognostic indicator for ASCVD patients, with its impact notable for its independence from eGFR, a variable that has traditionally dominated cardiovascular research. Despite its significance, UACR has yet to receive the attention it deserves within the current cardiovascular research paradigm. As an observational study associating risks, this research underscores the need for large-scale, prospective clinical trials to ascertain whether UACR is merely a risk marker for ASCVD or plays a more active role in its progression, and to evaluate whether interventions to reduce UACR levels could serve as an effective strategy in secondary prevention of ASCVD.

## Consent for publication

Informed consent was obtained from all individual participants included in the study. More detailed information can be found here: https://www.cdc.gov/nchs/nhanes/genetics/genetic_participants.htm

## Availability of data and material

Publicly available datasets were analyzed in this study. All original data can be found here: https://www.cdc.gov/nchs/nhanes. Additionally, the data supporting this study have been uploaded to Zenodo and can be accessed through the following link upon reasonable request: https://zenodo.org/records/14202427.

## Ethical approval and consent to participate

All procedures performed in studies involving human participants were following the ethical standards of the institutional and national research committee and with the 1964 Helsinki declaration and its later amendments or comparable ethical standards. More detailed information can be found here: https://www.cdc.gov/nchs/nhanes/irba98.htm

## Patient and public involvement

Patients and/or the public were not involved in the design, or conduct, or reporting, or dissemination plans of this research.

## Funding

This study was supported by the Zhejiang Administration Bureau of Traditional Chinese Medicine (2023ZR040 and 2024ZR144), the 10.13039/501100001809National Natural Science Foundation of China (62161160312), the Science Technology Department of Zhejiang Province (2020C03018), the Hangzhou Municipal Health Commission (Z20210019 and 20201203B178), the Zhejiang Medical Association Fund (2023ZYC-A13), the Hangzhou Biomedical and Health Industry Development Support Technology Special Project (2021WJCY002), the Clinical Medical Research Project of Zhejiang Medical Association (2018ZYC-A39), the Hangzhou TCM Hospital Affiliated to Zhejiang Chinese Medical University Hospital-Level Fund (YJ202305), and the 10.13039/501100004863Zhejiang Chinese Medical University School-Level Research Project (2024GJYY46). All sponsors mainly provide remuneration or gratuities for lectures, speeches, manuscript writing, educational activities, or rapid service fee, and do not play any role in study design, data collection, and analysis, or decisions to submit articles for publication.

## CRediT authorship contribution statement

**Houyong Zhu:** Writing – original draft, Funding acquisition. **Chao Yang:** Writing – original draft. **Xiao Liu:** Software, Formal analysis, Data curation. **Xiaoqun Xu:** Data curation. **Qilan Chen:** Writing – review & editing. **Xiaojiang Fang:** Writing – review & editing. **Jinyu Huang:** Methodology, Funding acquisition, Conceptualization. **Tielong Chen:** Methodology, Funding acquisition, Conceptualization.

## Declaration of competing interest

The authors declare the following financial interests/personal relationships which may be considered as potential competing interests:

Houyong Zhu reports administrative support and article publishing charges were provided by Zhejiang Administration Bureau of Traditional Chinese Medicine. Jinyu Huang reports administrative support and article publishing charges were provided by National Natural Science Foundation of China. Jinyu Huang reports administrative support and article publishing charges were provided by Science Technology Department of Zhejiang Province. Jinyu Huang reports administrative support and article publishing charges were provided by Hangzhou Municipal Health Commission. Tielong Chen reports administrative support and article publishing charges were provided by Zhejiang Medical Association Fund‌. Tielong Chen reports administrative support and article publishing charges were provided by Hangzhou Biomedical and Health Industry Development Support Technology Special Project. Tielong Chen reports administrative support and article publishing charges were provided by Clinical Medical Research Project of Zhejiang Medical Association. Houyong Zhu reports administrative support and article publishing charges were provided by Hangzhou TCM Hospital Affiliated to Zhejiang Chinese Medical University Hospital-Level Fund. Chao Yang reports administrative support and article publishing charges were provided by Zhejiang Chinese Medical University School-Level Research Project. If there are other authors, they declare that they have no known competing financial interests or personal relationships that could have appeared to influence the work reported in this paper.

## Appendix Supplementary materials

Image, application 1
