# Supplementary material for: Urinary albumin-to-creatinine ratio as an independent predictor of long-term mortality in atherosclerotic cardiovascular disease patients: A propensity score-matched study: UACR and Long-term Mortality in ASCVD
Source: Am J Prev Cardiol. 2024 Dec 18;21:100920. doi: 10.1016/j.ajpc.2024.100920 (PMC11728069; doi:10.1016/j.ajpc.2024.100920)
Supplement: Supplementary file 1 [file mmc1.docx]

**Supplementary Online Content**

**Catalogue**

[Appendix 1. Diagnostic Criteria for Diseases 3](#_Toc699655026)

[Supplementary Figure 1. Flow Diagram for Patient Recruitment 5](#_Toc1629054657)

[Supplementary Table 1. Comparison of Baseline Characteristics Between Excluded and Included Participants 6](#_Toc1252604596)

[Supplementary Table 2. Statistics of Missing Value and Extremesa 8](#_Toc743253431)

[Supplementary Table 3. Clinical Characteristics of Propensity Score Matched Patient Pairs Based on UACR Levels 9](#_Toc2095523865)

[Supplementary Table 4. All-Cause and Cardiovascular Mortality among Propensity Score Matched Patient Pairs 10](#_Toc737788255)

[Supplementary Figure 2. Subgroup Analyses for the Cardiovascular Mortality Across Across UACR Levels 11](#_Toc436624007)

[Supplementary Table 5. Sensitivity Analyses 12](#_Toc388063850)

## **Appendix 1. Diagnostic Criteria for Diseases**

**Chronic coronary syndrome**

The diagnostic criteria for chronic coronary syndrome refer to the 2019 ESC Guidelines for the diagnosis and management of chronic coronary syndromes (DOI: 10.1093/eurheartj/ehz425), which include the following populations: (1) suspected coronary heart disease, accompanied by stable angina symptoms and/or chest tightness; (2) New onset heart failure or left ventricular dysfunction may be coronary heart disease; (3) Less than 1 year after ACS or coronary revascularization, asymptomatic or asymptomatic stable; (4) >1 year after initial diagnosis or revascularization; (5) Suspect angina caused by vascular spasm or microvascular disease; (6) Asymptomatic coronary heart disease patients discovered during screening.

**Stroke**

Stroke is identified as a combination of standardized medical status questionnaires administered during self-reported physician diagnosis and personal interviews.

**Hypertension**

Includes history of hypertension diagnosed by the doctor, high systolic blood pressure (≥140mmHg) or high diastolic blood pressure (≥90mmHg), use of antihypertensive drugs, or final mortality due to hypertension.

**Diabetes**

Diabetes is identified to be diagnosed by a self-reported doctor, using insulin or oral hypoglycemic drugs, with fasting blood glucose ≥ 7.0mmol/L, or glycosylated hemoglobin ≥ 6.5%.

**Chronic lung disease**

Chronic lung disease is identified as a combination of standardized medical status questionnaires administered during self-reported physician diagnosis and personal interviews, which includes chronic bronchitis, bronchial asthma, and emphysema.

**Cancer**

Cancer is identified as a combination of standardized medical status questionnaires administered during self-reported physician diagnosis and personal interviews.

**Heart failure**

Heart failure is identified as a combination of standardized medical status questionnaires administered during self-reported physician diagnosis and personal interviews.

**Liver dysfunction**

Liver dysfunction is identified as a combination of standardized medical status questionnaires administered during self-reported physician diagnosis and personal interviews, and the value of alanine aminotransferase≥150U/L.

**Estimated glomerular filtration rate**

The estimatied glomerular filtration rate refers to the method of Inker et al (DOI: 10.1056/NEJMoa2102953). The specific formula is as follows:


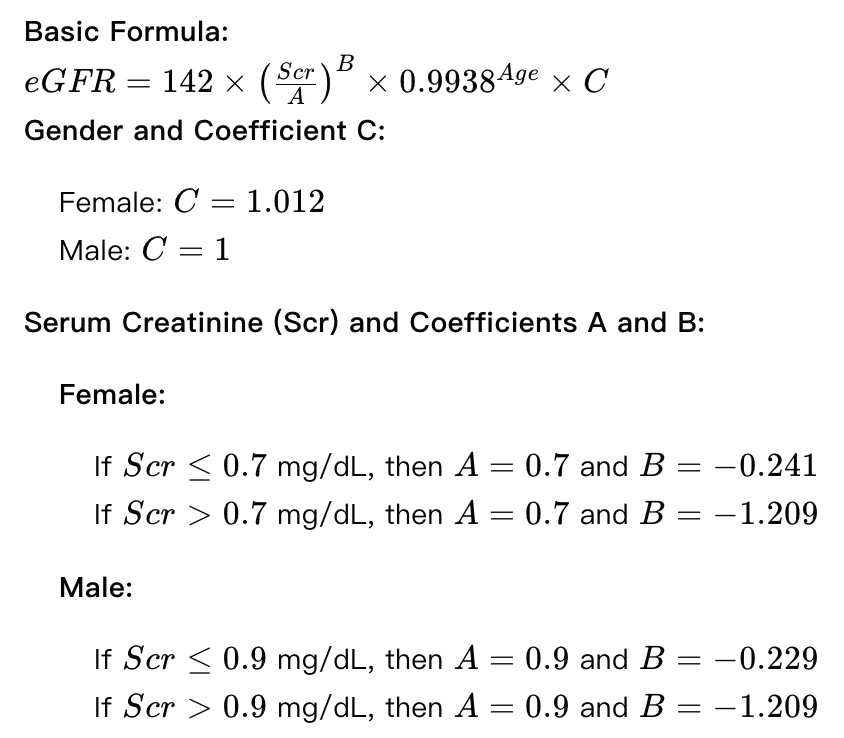


## **Supplementary Figure 1. Flow Diagram for Patient Recruitment**


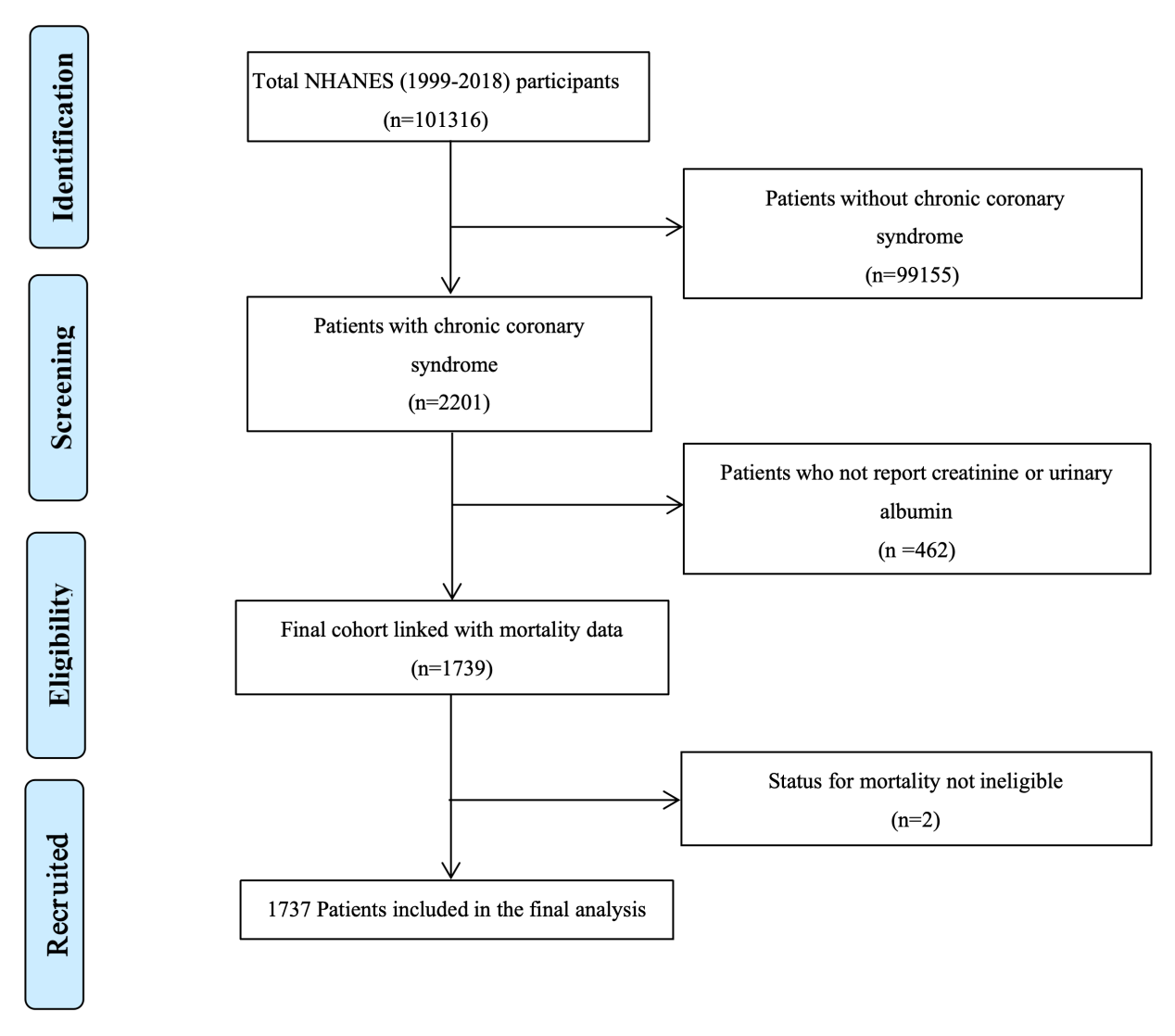


Supplementary Figure 1. Flow Diagram for Patient Recruitment.

## **Supplementary Table 1. Comparison of Baseline Characteristics Between Excluded and Included Participants**

| **Supplementary Table 1. Comparison of Baseline Characteristics Between Excluded and Included Participants** | | | |
| --- | --- | --- | --- |
| Vairable | Excluded Participants  (N=464) | Included Participants  (N=1737) | P Value |
| Age, median (quartile), years | 76 (64.25-83) | 70 (60-79) | <0.001 |
| Gender (Female), no. (%) | 231 (49.8) | 738 (42.5) | 0.005 |
| Race/ethnicity, no. (%) |  |  | 0.136 |
| Hispanic-Mexican American | 53 (11.4) | 224 (12.9) |  |
| Non-Hispanic White | 278 (59.9) | 1088 (62.6) |  |
| Non-Hispanic Black | 105 (22.6) | 311 (17.9) |  |
| Others^*^ | 28 (6.0) | 114 (6.6) |  |
| Education status, no. (%) |  |  | 0.144 |
| < High school | 190 (40.9) | 661 (38.1) |  |
| ≥ High school | 264 (56.9) | 1074 (61.8) |  |
| Marriage, no. (%) | 203 (43.8) | 960 (55.3) | <0.001 |
| Smoking, no. (%) |  |  | 0.035 |
| Never | 12 (2.6) | 42 (2.4) |  |
| Current | 42 (9.1) | 269 (15.5) |  |
| Former | 187 (40.3) | 758 (43.6) |  |
| Alcohol use, no. (%) | 38 (8.2) | 333 (19.2) | 0.184 |
| PIR, median (quartile) | 1.89 (1.17-3.28) | 1.86 (1.13-3.45) | 0.905 |
| BMI, median (quartile), kg/m^2^ | 28.70 (24.55-34.05) | 28.59 (25.28-32.78) | 0.986 |
| Tch, median (quartile), mmol/L | 4.62 (4.05-5.60) | 4.84 (4.11-5.69) | 0.247 |
| Diabetes, no. (%) | 148 (31.9) | 577 (33.2) | 0.591 |
| Hypertension, no. (%) | 353 (76.1) | 1404 (80.8) | 0.027 |
| Chronic lung disease, no. (%) | 117 (25.2) | 464 (26.7) | 0.511 |
| Liver dysfunction, no. (%) | 25 (5.4) | 104 (6.0) | 0.625 |
| Cancer, no. (%) | 113 (24.4) | 351 (20.2) | 0.053 |
| Family history of CVD, no. (%) | 59 (12.7) | 249 (14.3) | 0.399 |
| eGFR (mL/min/1.73m^2^) , no. (%) |  |  | <0.001 |
| >90 | 20 (4.3) | 472 (27.2) |  |
| 60-90 | 24 (5.2) | 763 (43.9) |  |
| 30-60 | 37 (8.0) | 443 (25.5) |  |
| 15-30 | 6 (1.3) | 50 (2.9) |  |
| <30 | 11 (2.4) | 9 (0.5) |  |
| Medication, no. (%) |  |  |  |
| ACEI or ARB | 181 (39.0) | 775 (44.6) | 0.046 |
| Beta-blocker | 178 (38.4) | 759 (43.7) | 0.053 |
| Lipid lowering therapy | 154 (33.2) | 810 (46.6) | <0.001 |
| Antiplatelet aggregation therapy | 75 (16.2) | 256 (14.7) | 0.362 |
| Calcium-channel blocker | 95 (20.5) | 295 (17.0) | 0.060 |

Supplementary Table 1. Comparison of Baseline Characteristics Between Excluded and Included Participants. ^*^This means "Other" encompasses both "Other Hispanic" and "Other Non-Hispanic Races". "Other Hispanic" refers to individuals who self-identify as Hispanic but are not of Mexican American descent. "Other Non-Hispanic Races" pertains to individuals who self-identify as a race other than white or black, or those who select multiple races. ACEI: angiotensin-converting enzyme inhibitor; ARB: angiotensin receptor blocker; BMI: body mass index; CVD: cardiovascular disease; eGFR: estimated glomerular filtration rate; PIR: poverty income ratio; Tch: total cholesterol; UACR: urinary albumin-to-creatinine ratio. Values are numbers (%) or medians (quartile).

## **Supplementary Table 2. Statistics of Missing Value and Extremesa**

| **Supplementary Table 2. Statistics of Missing Value and Extremesa** | | | | | | | |
| --- | --- | --- | --- | --- | --- | --- | --- |
|  | N | Mean | Std. Deviation | Missing | | No. of Extremes^a^ | |
|  |  |  |  | Count | Percent | Low | High |
| Age | 1737 | 67.82 | 13.006 | 0 | 0.0 | 19 | 0 |
| PIR | 1613 | 2.3302 | 1.48778 | 124 | 7.1 | 0 | 0 |
| BMI | 1654 | 29.5015 | 6.19195 | 83 | 4.8 | 0 | 41 |
| Tch | 1737 | 4.98483 | 1.254041 | 0 | 0.0 | 0 | 32 |
| Gender (Female) | 1737 |  |  | 0 | 0.0 |  |  |
| Race/ethnicity | 1737 |  |  | 0 | 0.0 |  |  |
| Education status | 1735 |  |  | 2 | 0.1 |  |  |
| Marriage | 1723 |  |  | 14 | 0.8 |  |  |
| Smoking | 1069 |  |  | 668 | 38.5 |  |  |
| Alcohol use | 1410 |  |  | 327 | 18.8 |  |  |
| Hypertension | 1736 |  |  | 1 | 0.1 |  |  |
| Diabetes | 1737 |  |  | 0 | 0.0 |  |  |
| Chronic lung disease | 1736 |  |  | 1 | 0.1 |  |  |
| Liver dysfunction | 1737 |  |  | 0 | 0.0 |  |  |
| Cancer | 1737 |  |  | 0 | 0.0 |  |  |
| Family history of CVD | 1069 |  |  | 668 | 38.5 |  |  |
| CKD | 1737 |  |  | 0 | 0.0 |  |  |
| UACR | 1737 |  |  | 0 | 0.0 |  |  |
| Antiplatelet aggregation therapy | 1584 |  |  | 153 | 8.8 |  |  |
| Beta-blocker | 1585 |  |  | 152 | 8.8 |  |  |
| ACEI or ARB | 1589 |  |  | 148 | 8.5 |  |  |
| Lipid lowering therapy | 1591 |  |  | 146 | 8.4 |  |  |
| a Number of cases outside the range (Q1 - 1.5*IQR, Q3 + 1.5*IQR). | | | | | | | |

Supplementary Table 2. Statistics of Missing Value and Extremesa. ACEI: angiotensin-converting enzyme inhibitor; ARB: angiotensin receptor blocker; BMI: body mass index; CKD: chronic kidney disease; CVD: cardiovascular disease; PIR: poverty income ratio; Tch: total cholesterol; UACR: urinary albumin-to-creatinine ratio.

## **Supplementary Table 3. Clinical Characteristics of Propensity Score Matched Patient Pairs Based on UACR Levels**

| **Supplementary Table 3. Clinical Characteristics of Propensity Score Matched Patient Pairs Based on UACR Levels** | | | | | | | | |
| --- | --- | --- | --- | --- | --- | --- | --- | --- |
| Vairable | UACR with a cutoff of 30mg/g | | | | UACR with a cutoff of 300mg/g | | | |
|  | 0 (≤30)  (N=444) | 1 (>30)  (N=444) | P Value | Standardized  Difference | 0 (≤300)  (N=109) | 1 (>300)  (N=109) | P Value | Standardized Difference |
| Age, median (quartile), years | 73 (64-80) | 71.5 (64-80) | 0.948 | -0.017 | 71 (62.5-79) | 69 (61-80) | 0.667 | -0.069 |
| Gender (Female), no. (%) | 174 (39.2) | 176 (39.6) | 0.891 | 0.009 | 49 (45.0) | 40 (36.7) | 0.215 | -0.171 |
| Race/ethnicity, no. (%) |  |  | 0.618 |  |  |  | 0.773 |  |
| Hispanic-Mexican American | 61 (13.7) | 63 (14.2) |  | - | 17 (15.6) | 23 (21.1) |  | - |
| Non-Hispanic White | 274 (61.7) | 268 (60.4) |  | -0.028 | 63 (57.8) | 59 (54.1) |  | -0.073 |
| Non-Hispanic Black | 93 (20.9) | 89 (20) |  | -0.022 | 24 (22.0) | 22 (20.2) |  | -0.043 |
| Others^*^ | 16 (3.6) | 24 (5.4) |  | 0.083 | 5 (4.6) | 5 (4.6) |  | 0.000 |
| Education status, no. (%) |  |  | 0.494 |  |  |  | 0.335 |  |
| < High school | 186 (41.9) | 195 (43.9) |  | - | 41 (37.6) | 48 (44.0) |  | -0.129 |
| ≥ High school | 258 (58.1) | 248 (55.9) |  | -0.045 | 68 (62.4) | 61 (56.0) |  | - |
| Marriage, no. (%) | 223 (50.2) | 225 (50.7) | 0.991 | 0.009 | 52 (47.7) | 53 (48.6) | 0.892 | 0.018 |
| Smoking, no. (%) |  |  | 0.817 |  |  |  | 0.863 |  |
| Never | 8 (1.8) | 9 (2.0) |  | - | 4 (3.7) | 4 (3.7) |  |  |
| Current | 63 (14.2) | 59 (13.3) |  | -0.027 | 18 (16.5) | 17 (15.6) |  | -0.026 |
| Former | 212 (47.7) | 202 (45.5) |  | -0.045 | 37 (33.9) | 43 (39.4) |  | 0.112 |
| Alcohol use, no. (%) | 83 (18.7) | 77 (17.3) | 0.780 | -0.035 | 14 (12.8) | 23 (21.1) | 0.263 | 0.197 |
| PIR, median (quartile) | 1.85 (1.16-2.83) | 1.89 (1.18-2.65) | 0.947 | -0.011 | 1.89 (1.12-3.00) | 1.95 (1.11-2.70) | 0.845 | 0.003 |
| BMI, median (quartile), kg/m^2^ | 29.37 (26.21-33.32) | 28.90 (25.26-32.92) | 0.228 | -0.072 | 28.97 (25.84-31.70) | 28.07 (25.36-32.30) | 0.921 | 0.047 |
| Tch, median (quartile), mmol/L | 4.84 (4.06-5.79) | 4.86 (4.12-5.71) | 0.702 | 0.023 | 4.94 (4.23-6.03) | 5.04 (4.34-6.01) | 0.546 | 0.104 |
| Diabetes, no. (%) | 206 (46.4) | 210 (47.3) | 0.788 | 0.018 | 68 (62.4) | 71 (65.1) | 0.673 | 0.058 |
| Hypertension, no. (%) | 381 (85.8) | 375 (84.5) | 0.571 | -0.038 | 98 (89.9) | 97 (89.0) | 0.826 | -0.029 |
| Chronic lung disease, no. (%) | 118 (26.6) | 108 (24.3) | 0.441 | -0.053 | 21 (19.3) | 23 (21.1) | 0.736 | 0.044 |
| Liver dysfunction, no. (%) | 34 (7.7) | 31 (7.0) | 0.699 | -0.026 | 5 (4.6) | 5 (4.6) | 1.000 | 0.000 |
| Cancer, no. (%) | 108 (24.3) | 99 (22.3) | 0.775 | -0.049 | 25 (22.9) | 24 (22) | 0.871 | -0.022 |
| Family history of CVD, no. (%) | 67 (15.1) | 61 (13.7) | 0.847 | -0.040 | 11 (10.1) | 12 (11.0) | 0.956 | 0.030 |
| eGFR (mL/min/1.73m^2^) , no. (%) |  |  | 0.807 |  |  |  | 0.956 |  |
| >90 | 85 (19.1) | 89 (20.0) |  | - | 16 (14.7) | 20 (18.3) |  | - |
| 60-90 | 197 (44.4) | 182 (41.0) |  | -0.069 | 29 (26.6) | 28 (25.7) |  | -0.022 |
| 30-60 | 147 (33.1) | 160 (36.0) |  | 0.062 | 48 (44.0) | 47 (43.1) |  | -0.018 |
| 15-30 | 13 (2.9) | 12 (2.7) |  | -0.009 | 12 (11.0) | 11 (10.1) |  | -0.026 |
| <30 | 2 (0.5) | 1 (0.2) |  | -0.019 | 4 (3.7) | 3 (2.8) |  | 0.051 |
| Medication, no. (%) |  |  |  |  |  |  |  |  |
| ACEI or ARB | 220 (49.5) | 214 (48.2) | 0.919 | -0.027 | 69 (63.3) | 63 (57.8) | 0.701 | -0.110 |
| Beta-blocker | 212 (47.7) | 208 (46.8) | 0.941 | -0.018 | 56 (51.4) | 58 (53.2) | 0.963 | 0.037 |
| Lipid lowering therapy | 214 (48.2) | 199 (44.8) | 0.594 | -0.068 | 52 (47.7) | 56 (51.4) | 0.861 | 0.073 |
| Antiplatelet aggregation therapy | 64 (14.4) | 64 (14.4) | 1.000 | 0.000 | 20 (18.3) | 19 (17.4) | 0.984 | -0.024 |
| Calcium-channel blocker | 87 (19.6) | 100 (22.5) | 0.560 | 0.070 | 28 (25.7) | 26 (23.9) | 0.952 | -0.043 |

Supplementary Table 3. Clinical Characteristics of Propensity Score Matched Patient Pairs Based on UACR Levels. ^*^This means "Other" encompasses both "Other Hispanic" and "Other Non-Hispanic Races". "Other Hispanic" refers to individuals who self-identify as Hispanic but are not of Mexican American descent. "Other Non-Hispanic Races" pertains to individuals who self-identify as a race other than white or black, or those who select multiple races. ACEI: angiotensin-converting enzyme inhibitor; ARB: angiotensin receptor blocker; BMI: body mass index; CVD: cardiovascular disease; eGFR: estimated glomerular filtration rate; PIR: poverty income ratio; Tch: total cholesterol; UACR: urinary albumin-to-creatinine ratio. Values are numbers (%) or medians (quartile).

## **Supplementary Table 4. All-Cause and Cardiovascular Mortality among Propensity Score Matched Patient Pairs**

| **Supplementary Table 4. All-Cause and Cardiovascular Mortality among Propensity Score Matched Patient Pairs** | | | | |
| --- | --- | --- | --- | --- |
| Outcomes | UACR with a cutoff of 30mg/g, HR (95% Cl) | | UACR with a cutoff of 300mg/g, HR (95% Cl) | |
|  | 0 (≤30)  (N=444) | 1 (>30)  (N=444) | 0 (≤300)  (N=109) | 1 (>300)  (N=109) |
| All-cause mortality | 1.000 (Reference) | 1.468 (1.254-1.719) | 1.000 (Reference) | 1.935 (1.399-2.675) |
| P Value |  | <0.001 |  | <0.001 |
| Cardiovascular mortality | 1.000 (Reference) | 1.963 (1.499-2.572) | 1.000 (Reference) | 1.799 (1.074-3.013) |
| P Value |  | <0.001 |  | 0.026 |

Supplementary Table 4. All-Cause and Cardiovascular Mortality among Propensity Score Matched Patient Pairs. CI: confidence interval, eGFR: estimated glomerular filtration rate; UACR: Urinary albumin-to-creatinine ratio, HR: hazard ratio.

## **Supplementary Figure 2. Subgroup Analyses for the Cardiovascular Mortality Across Across UACR Levels**


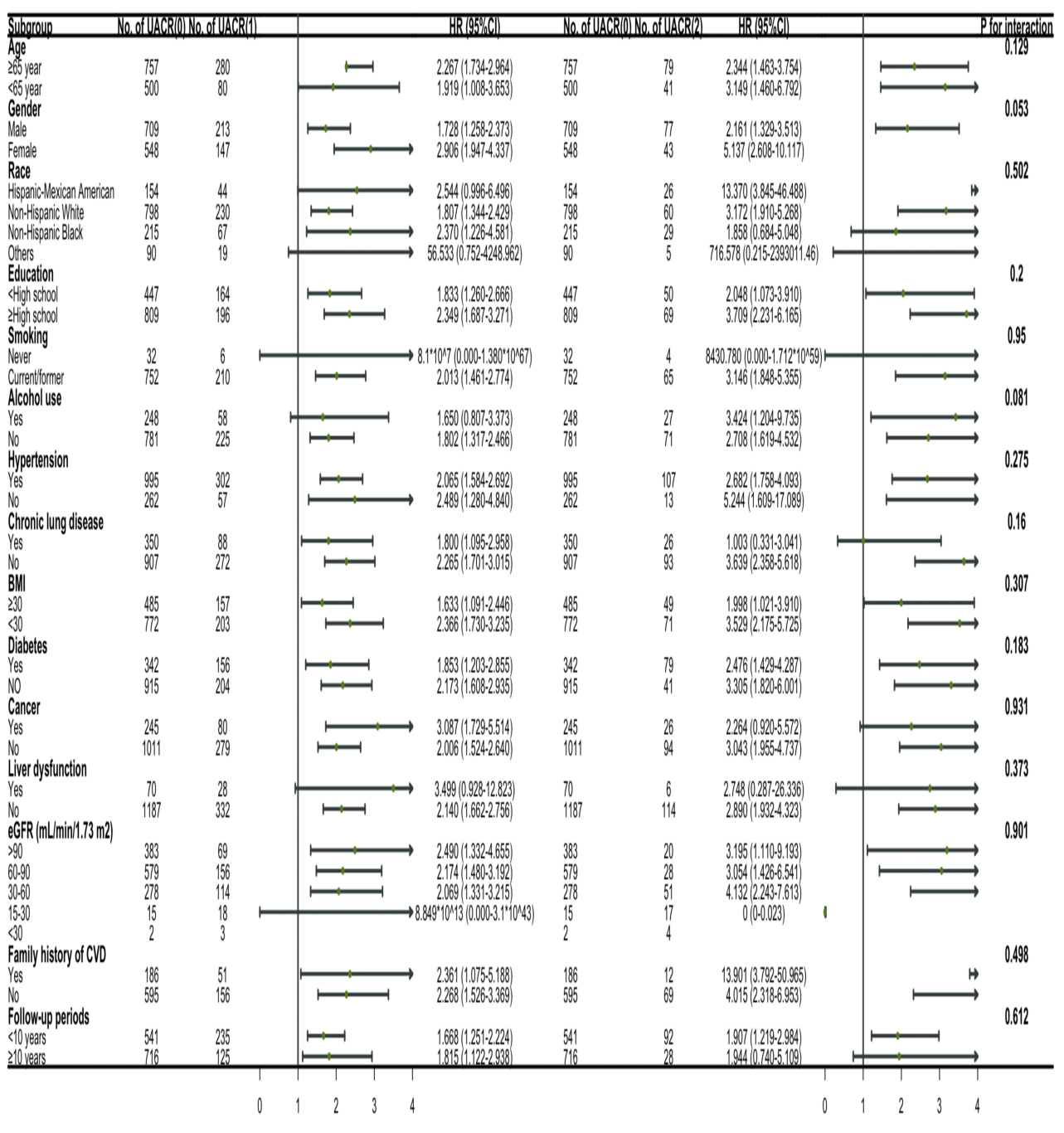


Supplementary Figure 2. Subgroup Analyses for the Cardiovascular Mortality Across Across UACR Levels. BMI: body mass index; CVD: cardiovascular disease; eGFR: estimated glomerular filtration rate; UACR: urinary albumin-to-creatinine ratio, HR: hazard ratio.

## **Supplementary Table 5. Sensitivity Analyses**

| **Supplementary Table 5. Sensitivity Analyses** | | | | | | | | | | |
| --- | --- | --- | --- | --- | --- | --- | --- | --- | --- | --- |
| Outcomes | CCS,  HR (95% Cl) | P Value | Stroke,  HR (95% Cl) | P Value | HF,  HR (95% Cl) | P Value | Non-HF,  HR (95% Cl) | P Value | TP^*^,  HR (95% Cl) | P Value |
| All-cause mortality |  |  |  |  |  |  |  |  |  |  |
| UACR |  |  |  |  |  |  |  |  |  |  |
| 0 | 1.000 (Reference) |  | 1.000 (Reference) |  | 1.000 (Reference) |  | 1.000 (Reference) |  | 1.000 (Reference) |  |
| 1 | 1.620 (1.371-1.914) | <0.001 | 1.434 (1.122-1.833) | 0.004 | 1.635 (1.240-2.157) | <0.001 | 1.584 (1.323-1.898) | <0.001 | 1.483 (1.158-1.901) | 0.002 |
| 2 | 2.210 (1.690-2.891) | <0.001 | 2.931 (1.991-4.316) | <0.001 | 2.321 (1.503-3.583) | <0.001 | 2.388 (1.795-3.176) | <0.001 | 3.061 (2.097-4.469) | <0.001 |
| P for trend | <0.001 |  | <0.001 |  | <0.001 |  | <0.001 |  | <0.001 |  |
| Cardiovascular mortality |  |  |  |  |  |  |  |  |  |  |
| UACR |  |  |  |  |  |  |  |  |  |  |
| 0 | 1.000 (Reference) |  | 1.000 (Reference) |  | 1.000 (Reference) |  | 1.000 (Reference) |  | 1.000 (Reference) |  |
| 1 | 2.090 (1.590-2.749) | <0.001 | 2.036 (1.335-3.103) | <0.001 | 2.148 (1.391-3.319) | <0.001 | 1.961 (1.437-2.675) | <0.001 | 1.812 (1.220-2.692) | 0.003 |
| 2 | 2.347 (1.482-3.717) | <0.001 | 6.204 (3.168-12.150) | <0.001 | 3.137 (1.600-6.148) | <0.001 | 2.910 (1.765-4.798) | <0.001 | 3.500 (1.826-6.708) | <0.001 |
| P for trend | <0.001 |  | <0.001 |  | <0.001 |  | <0.001 |  | <0.001 |  |

Supplementary Table 5. Sensitivity Analyses. ^*^Indicates that these patients, in addition to receiving lipid-lowering therapy, are also on at least one other form of secondary prevention medication, including antiplatelet aggregation therapy, beta-blockers, calcium channel blockers, angiotensin-converting enzyme inhibitors, or angiotensin receptor blockers. CCS, chronic coronary syndrome, CI: confidence interval, HF: heart failure, HR: hazard ratio, TP: treated population, UACR: urinary albumin-to-creatinine ratio.
